# A Theoretical Approach for the Electrochemical Characterization of Ciliary Epithelium

**DOI:** 10.3390/life10020008

**Published:** 2020-01-23

**Authors:** Riccardo Sacco, Giovanna Guidoboni, Joseph W. Jerome, Giulio Bonifazi, Nicholas M. Marazzi, Alice C. Verticchio Vercellin, Matthew S. Lang, Alon Harris

**Affiliations:** 1 Dipartimento di Matematica, Politecnico di Milano, 20133 Milano MI, Italy; 2 Department of Electrical Engineering and Computer Science, University of Missouri, Columbia, MO 65211, USA; guidobonig@missouri.edu (G.G.); marazzin@mail.missouri.edu (N.M.M.); 3 Department of Mathematics, University of Missouri, Columbia, MO 65211, USA; 4 Department of Mathematics, George Washington University, Washington, DC 20052, USA; jwj620@email.gwu.edu; 5 Basque Center for Applied Mathematics, Bilbao 48009, Spain; gbonifazi@bcamath.org; 6 Department of Ophthalmology, Icahn School of Medicine at Mount Sinai Hospital, New York, NY 10029, USA; alice.verticchio@gmail.com (A.C.V.V.); palonharris@gmail.com (A.H.); 7 Eye Clinic, University of Pavia, 27100 Pavia, Italy; 8 Scientific Institute for Research, Hospitalization and Healthcare(IRCCS) - Fondazione Bietti, 00198 Rome, Italy; 9 School of Medicine, Indiana University, Indianapolis, IN 47405, USA; mslang@iu.edu

**Keywords:** aqueous humor, ciliary epithelium, ultrafiltration, ionic secretion, ion transporters, physiology, electrochemical conditions, fluid-dynamical conditions, mathematical model, simulation

## Abstract

The ciliary epithelium (CE) is the primary site of aqueous humor (AH) production, which results from the combined action of ultrafiltration and ionic secretion. Modulation of ionic secretion is a fundamental target for drug therapy in glaucoma, and therefore it is important to identify the main factors contributing to it. As several ion transporters have been hypothesized as relevant players in CE physiology, we propose a theoretical approach to complement experimental methods in characterizing their role in the electrochemical and fluid-dynamical conditions of CE. As a first step, we compare two model configurations that differ by *(i)* types of transporters included for ion exchange across the epithelial membrane, and by *(i)* presence or absence of the intracellular production of carbonic acid mediated by the carbonic anhydrase enzyme. The proposed model configurations do not include neurohumoral mechanisms such as P2Y receptor-dependent, cAMP, or calcium-dependent pathways, which occur in the ciliary epithelium bilayer and influence the activity of ion transporters, pumps, and channels present in the cell membrane. Results suggest that one of the two configurations predicts sodium and potassium intracellular concentrations and transmembrane potential much more accurately than the other. Because of its quantitative prediction power, the proposed theoretical approach may help relate phenomena at the cellular scale, that cannot be accessed clinically, with phenomena occurring at the scale of the whole eye, for which clinical assessment is feasible.

## 1. Introduction

The last ten years have witnessed a continuous increase in the development and use of theoretical investigation tools based on mathematical models and numerical algorithms for computer simulation in Ophthalmology (see [1] and references cited therein). Motivated by the need of providing a multiscale and multiphysics platform capable of capturing the complex interplay among ocular biomechanics, fluid dynamics, hemodynamics, and electrochemistry governing ocular physiology, our research team has been pursuing the general aim of devising and implementing a patient-specific mathematical virtual simulator for the eye [2,3,4].

In this paper we focus our attention on a specific region of the eye, the ciliary body, and, in particular, we investigate the physiology of ciliary epithelium (CE) and the mechanisms underlying the production of aqueous humor (AH). This choice is motivated by the fact that AH flow and its regulation significantly contribute to determining the level of intraocular pressure (IOP) [5,6]. Elevated IOP is the only approved treatable risk factor in glaucoma, an optic neuropathy affecting millions of people worldwide, that is characterized by a progressive degeneration of retinal ganglion cells which ultimately leads to irreversible vision loss [7,8]. AH production strongly relies on osmotic pressure gradients that are generated across the CE by active ionic secretion, which represents a fundamental target for drug therapy in glaucoma. Since several ion transporters have been hypothesized as important players in the ionic secretion across the CE, we propose in the next sections a theoretical approach to help characterize their role.

More specifically, we first provide, (*i*), a characterization of the scale levels that are present in the eye system; then, (*ii*), we focus on the physiology of the ciliary epithelium, which is the region of the eye where regulation of aqueous humor production occurs; finally, (*iii*), we discuss the connection between aqueous humor production and ocular physiology. This connection provides the ultimate motivation to the development of a sound theoretical model of the ciliary epithelium and aqueous humor production that is capable to account for the principal scale levels and related biophysical mechanisms, namely:

1. the flow of ions through ion transporters and the combined effect of channels and pumps (membrane scale level);
2. the overall contribution to AH production by a single cell of the ciliary epithelium (cellular scale level);
3. the hydrostatic and oncotic pressure effects (eye scale level).

To achieve this ambitious goal, the first step is to find a suitable model for each scale level, and then to characterize its physiological baseline conditions. The first contribution in this direction has been made in [9] where a baseline characterization of parameters for AH flow at the eye level was performed by means of an equivalent electric circuit. To the best of our knowledge, however, no investigation has been made to characterize the electrochemical baseline conditions for the cellular level. This is the main objective of the present article and will be obtained by combining the action of multiple channels and pumps in the ciliary epithelium and studying their functionality and interaction with the intracellular environment. Future steps will be to utilize the model for the comparative study of different scenarios, including the effect of drugs or patient-specific features.

The mathematical model proposed in this article is the first step towards a deep, physically-based, investigation of the electrophysiology of the ciliary epithelium whose ultimate goal is to shed light on the complex mechanisms regulating the process of AH formation in the ciliary body of the human eye. As such, the model is affected by several limitations, particularly, it does not account for neurohumoral mechanisms such as P2Y receptor-dependent, cAMP or calcium-dependent pathways, which occur in the ciliary epithelium bilayer and have been recognized to influence the activity of ion transporters, pumps and channels present in the cell membrane (see [10] and references cited therein and the experimental studies illustrated in [11,12]). More details on how to extend the present model to include neurohumoral intracellular mechanisms will be given in Section 5.

### 1.1. The Multiscale Architecture of Aqueous Humor Production

**Figure 1:**
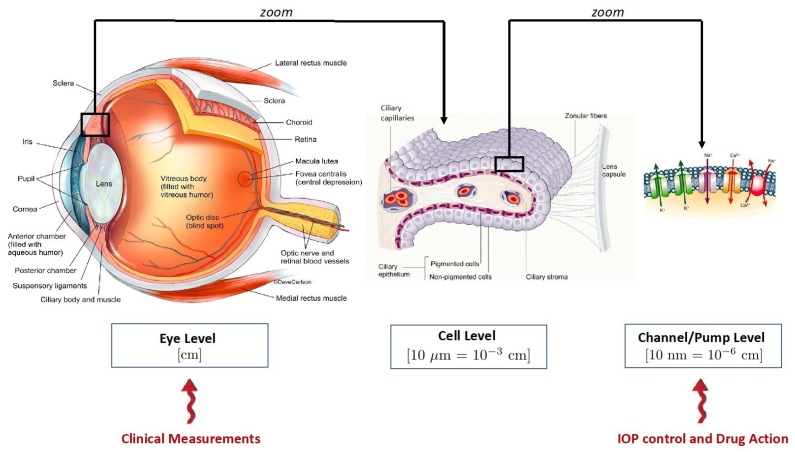
Multiscale architecture of aqueous humor production. (**Left panel**) Macroscopic scale, which is the level of the whole eye (image taken from [13]). (**Central panel**) Cellular scale, which is the level of the pigmented and nonpigmented epithelial cells (image taken from [14]). (**Right panel**) Cellular membrane scale, which is the level of the membrane of the nonpigmented epithelial cells (image taken from [15]).

The study of AH production naturally requires a multiscale approach, as schematically represented in Figure 1. Three scales are identified in the scheme:

**(a)**  the macroscopic scale. This is the level of observation corresponding to the eye globe and its characteristic length is in the order of centimeters;
**(b)**  the cellular scale. This is the level of observation corresponding to the PEC/NPEC couplet and its characteristic length is in the order of tens of microns (i.e., $10^{-3}\;\mathrm{cm}$);
**(c)**  the membrane scale. This is the level of observation corresponding to the membrane of the NPEC and its characteristic length is in the order of tens of nanometers (i.e., $10^{-6}\;\mathrm{cm}$).

It is important to notice that, regardless of the huge range of characteristic lengths in the AH production system, only the macroscopic scale can be experimentally accessed in a non-invasive manner and with a relatively low effort. Experimental studies at the cellular and membrane scales are much more challenging and scarce than those at the macroscopic scale, thereby limiting the quantity and quality of data available for a comprehensive assessment of AH production in health and diseases. In this perspective, mathematical modeling can help establish mechanistic links across scales and simulate “invisible” microscale phenomena that, though, may play a major role in determining the biophysical properties of the eye, ultimately manifesting into the function of vision. In particular, the present contribution focuses on the cellular scale and complements our ongoing work on the eye level [3] and channel/pump level [16,17,18]. The long term goal is to ultimately combine these scales within a unified modeling framework to provide a virtual simulation tool capable of producing a theoretical synopsis of patient conditions to be confronted with clinical measurements.

### 1.2. Ciliary Epithelium and Aqueous Humor Production

**Figure 2:**
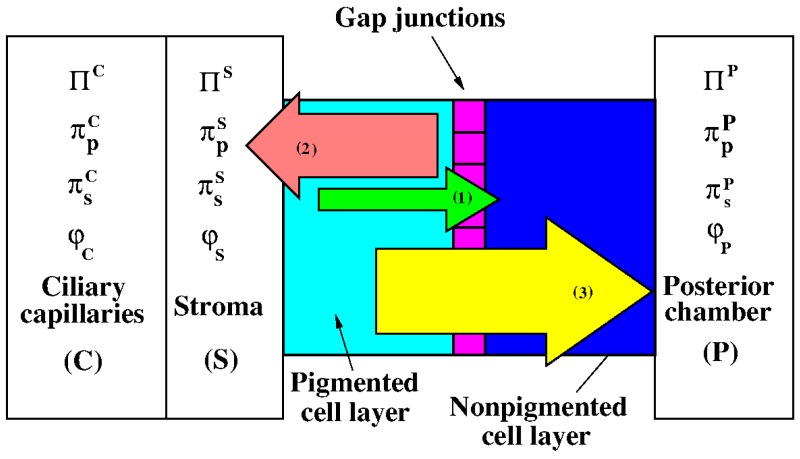
A schematic representation of the biophysical mechanisms that underlie AH production at the cell level. A couplet of pigmented/non-pigmented epithelial cells, including also gap junctions, is located between ciliary capillaries (*C*) and stroma (*S*), see the left side of the figure, and posterior chamber (*P*), see right side of the figure. For X=C,S,P, we consider the following biophysical quantities: hydrostatic pressure ($\Pi^{X}$), oncotic pressure ($\pi_{p}^{X}$), osmotic pressure ($\pi_{s}^{X}$), and electric potential ($\varphi_{X}$). Typically, $\Pi^{C}>\Pi^{S}>\Pi^{P}$, with $\Pi^{P}$ equal to the intraocular pressure (IOP), $\pi_{p}^{C}>\pi_{p}^{S}>\pi_{p}^{P}$ and $\pi_{s}^{S}=\pi_{s}^{C}<\pi_{s}^{P}$. As a consequence, hydrostatic pressure difference (1, light green arrow), oncotic pressure difference (2, pink arrow), and osmotic pressure difference (3, yellow arrow) are established across the ciliary epithelium. Arrow sizes provide an indication of the magnitude of each contribution to AH production.

The ciliary epithelium (CE) is the primary site of aqueous humor (AH) production. Figure 2 provides a schematic view of one CE cell couplet, comprising a pigmented epithelial cell and a non-pigmented epithelial cell, together with the ciliary capillaries, the ciliary stroma, the posterior chamber and the mechanisms which concur to determine AH flow across the cell couplet.

Following [19], the process of AH production consists of three stages:

1. Convective delivery of water, ions, proteins, and metabolic fuel by the ciliary circulation;
2. Ultrafiltration of water and ions (driven by oncotic and hydrostatic pressure gradients) and diffusion of larger molecules from the capillaries into the stroma (driven by concentration gradients);
3. Active ionic secretion into the basolateral space between the NPE cells which promotes water flow down the resulting osmotic gradient.

The volumetric flow rate $J_{AH}^{uf}$ of aqueous humor due to ultrafiltration is given by the following relation

(1) $$\begin{array}{ccc} \; & J_{AH}^{uf}=J_{AH}^{uf,hyd}+J_{AH}^{uf,onc}, & \end{array}$$

where:

1. $J_{AH}^{uf,hyd}$: volumetric flow rate of aqueous humor due to the difference between the hydrostatic pressure $\Pi^{C}$ in the ciliary capillaries and the hydrostatic pressure $\Pi^{P}$ in the posterior chamber assumed to be equal to the intraocular pressure (IOP)
  (2) $$\begin{array}{ccc} \; & \Delta \pi_{hyd}:=\Pi^{C}-\Pi^{P}; & \end{array}$$
2. $J_{AH}^{uf,onc}$: volumetric flow rate of aqueous humor due to the difference between the oncotic pressure $\pi_{p}^{C}$ in the ciliary capillaries and the oncotic pressure $\pi_{p}^{P}$ in the posterior chamber
  (3) $$\begin{array}{ccc} \; & \Delta \pi_{p}:=\pi_{p}^{C}-\pi_{p}^{P}. & \end{array}$$

The mathematical characterization of the two contributions in (1) can be expressed by the following formulas (see [19]):

(4) $$\begin{array}{ccc} \; & J_{AH}^{uf,hyd}=L_{p}\Delta \pi_{hyd}=L_{p}\left(\Pi^{C}-\Pi^{P}\right), & \end{array}$$

(5) $$\begin{array}{ccc} \; & J_{AH}^{uf,onc}=-L_{p}\sigma_{p}\Delta \pi_{p}=-L_{p}\sigma_{p}\left(\pi_{p}^{C}-\pi_{p}^{P}\right), & \end{array}$$

where $L_{p}$ and $\sigma_{p}$ are the filtration coefficient and protein reflection coefficient of the NPE bilayer, respectively. Since $\Pi^{C}$ is usually larger than $\Pi^{P}$, the hydrostatic pressure gradient (proportional to $\Delta \pi_{hyd}$) is directed from the ciliary capillaries into the posterior chamber (arrow (1) in Figure 2) and $J_{AH}^{uf,hyd}>0$. Conversely, as there is a lack of proteins in the posterior chamber, approximated as $\pi_{p}^{P}=0$, the oncotic pressure gradient (proportional to $-\Delta \pi_{p}$) is directed from the posterior chamber into the ciliary capillaries (arrow (2) in Figure 2) and $J_{AH}^{uf,onc}<0$. In [19], Kiel et al. report that, in physiological conditions, $\Delta \pi_{hyd}\approx 5$ mmHg and $\Delta \pi_{p}\approx 20$ mmHg, meaning that AH would move back into the stroma if only passive mechanisms (i.e., driven by hydrostatic and oncotic pressure differences) are taken into account. However, physiological evidence shows that AH flows in the opposite direction, from the capillary circulation into the posterior chamber, to be subsequently drained out of the eye by conventional trabecular meshwork pathway, which is believed to be the major route of AH drainage, and uveoscleral outflow pathway (see [20]). Therefore, a third mechanism must be responsible for AH production towards the posterior chamber. This mechanism relies on the difference between the osmotic pressure $\pi_{s}^{P}$ in the posterior chamber and the osmotic pressure $\pi_{s}^{S}$ in the stroma (that is assumed to coincide with the osmotic pressure in the ciliary capillaries)

(6) $$\begin{array}{ccc} \; & \Delta \pi_{s}:=\pi_{s}^{P}-\pi_{s}^{S}. & \end{array}$$

The osmotic pressure $\pi_{s}$ of a solute *s* with molar concentration $n_{s}$ (units: $\mathrm{mol}\;m^{-3}=\mathrm{mM}$) is defined by the van’t Hoff formula:

(7) $$\begin{array}{ccc} \; & \pi_{s}=\kappa n_{s}RT, & \end{array}$$

where $R=8.3144508\;{\mathrm{Jmol}}^{-1}K^{-1}$ is the gas constant, *T* is the temperature measured in K and κ is a dimensionless parameter referred to as the van’t Hoff factor, which is taken to equal to 1 henceforth. The osmotic pressure difference $\Delta \pi_{s}$ is represented by the yellow arrow (3) in Figure 2 and its contribution makes the AH production an active process requiring the expenditure of metabolic energy. The volumetric flow rate $J_{AH}^{ion,sec}$ of aqueous humor due to active ionic secretion from the NPE cells into the posterior chamber can be expressed by the following relation (see [19])

(8) $$\begin{array}{ccc} \; & J_{AH}^{ion,sec}=L_{s}\Delta \pi_{s}=L_{s}\left(\pi_{s}^{P}-\pi_{s}^{S}\right). & \end{array}$$

where $L_{s}$ is the osmotic filtration coefficient of the NPE bilayer. Combining ultrafiltration and active ionic secretion yields the following mathematical characterization of the AH production process

(9) $$\begin{array}{ccc} \; & J_{AH}=J_{AH}^{uf}+J_{AH}^{ion,sec}=L_{p}\left[\left(\Pi^{C}-\Pi^{P}\right)-\sigma_{p}\left(\pi_{p}^{C}-\pi_{p}^{P}\right)\right]+L_{s}\left(\pi_{s}^{P}-\pi_{s}^{S}\right). & \end{array}$$

Let us assume throughout the remainder of the article that T=310.15K (corresponding to 37 °C). The conversion from molar concentrations $n_{s}^{P}$ and $n_{s}^{S}$ into osmotic pressure using the van’t Hoff Equation (7) yields $\pi_{s}^{P}=2843$ mmHg and $\pi_{s}^{S}=2804.4$ mmHg. Plugging back these quantities and the remaining values of the parameters listed in Table 1 into (9) we obtain

(10) $$\begin{array}{ccc} \; & J_{AH}^{uf,hyd}=2\cdot 10^{-4}\;\mathrm{mL}\;\min^{-1},\;J_{AH}^{uf,onc}=-3.974\cdot 10^{-4}\;\mathrm{mL}\;\min^{-1} & \\ \; & \Rightarrow J_{AH}^{uf}=2\cdot 10^{-4}-3.974\cdot 10^{-4}=-1.974\cdot 10^{-4}\;\mathrm{mL}\;\min^{-1}, & \end{array}$$

which shows that the sole passive mechanisms give rise to a AH flow back into the stroma and not into the posterior chamber. However, the highly positive active pressure difference $\pi_{s}^{P}-\pi_{s}^{S}=38.681$ mmHg compared to the negative passive pressure difference $\left(\Pi^{C}-\Pi^{P}\right)-\sigma_{p}\left(\pi_{p}^{C}-\pi_{p}^{P}\right)=-9.8701$ mmHg gives rise to a volumetric flow rate into the posterior chamber $J_{AH}^{ion,sec}=5.9354\cdot 10^{-3}\;\mathrm{mL}\;\min^{-1}$, which more than compensates the back flow of AH in (10), and results in a volumetric flow rate $J_{AH}=5.738\cdot 10^{-3}\;\mathrm{mL}\;\min^{-1}$ from the stroma into the posterior chamber.

Despite the fact that active secretion is the main player in AH production, a clear understanding of the physiology of AH secretion is still lacking, particularly because there is no consensus on which ion transporters are located on the cellular membranes of CE and which of them are the more active (see [19,21,22,23,24]). A brief overview of the current literature in the field is provided in the next section.

### 1.3. Active Secretion of Aqueous Humor

Active transport of ions across the ciliary epithelium provides the driving force for aqueous humor production and is dependent upon the interplay between the ion transporters and channels located on the membranes of the PE and NPE. Conceptual models (see [25], Chapter 1) describing the differential location and role of these transporters and channels have been proposed in [23,26,27]. While no single conceptual model has been adopted as the current standard, it is well established that ion transport is a sequential process involving facilitated cotransport of ions across the PE basolateral membrane, diffusion of ions into NPE cells via gap junctions at the apical junction, and a combination of primary active transport and facilitated diffusion in order to shift ions across the NPE basolateral membrane and into the posterior chamber [24]. It is widely accepted that:

1. Proteins are practically absent inside the CE cells. As a consequence, HCO${}_{3}^{-}$ is the main responsible for maintaining the pH of the cells within physiological values (between 7.21 and 7.4, see [27]);
2. The Na${}^{+}$/K${}^{+}$-ATPase pump (shortly, Na${}^{+}$/K${}^{+}$ pump) is essential to set the cell off its electrochemical balance and create a driving force for the secretion of AH. Studies show that inhibiting this pump results in blocking AH secretion (see [28])
3. Elevated K${}^{+}$ concentrations on the aqueous side (the posterior chamber in the scheme of Figure 2) lead to a significant depolarization of the nonpigmented epithelial cells (see [29]);
4. Carbonic Anhydrase (CA) plays a major role in bicarbonate regulation inside the CE [30] so that Carbonic Anhydrase Inhibitors (CAI) are commonly utilized as IOP-lowering drugs [6].

An overview of the current understanding of ion transporters and channels and their localization on the membrane of the ciliary epithelium is summarized in Table 2.

A variety of cotransporter mechanisms have been identified on the basolateral membrane of the PE including Na${}^{+}$/K${}^{+}$/2Cl${}^{-}$ symport, Na${}^{+}$/H^+^ antiport, Cl${}^{-}$/HCO${}_{3}^{-}$ antiport, and Na${}^{+}$/HCO${}_{3}^{-}$ symport (see [32,36,38,39,42,43,44,45]). Intracellular transport of ions within and between the PE and NPE layers is facilitated by gap junctions localized to the apical and lateral membranes of both layers [31]. With regards to ion transport across the NPE into the posterior chamber, multiple studies have confirmed the presence of Na${}^{+}$/K${}^{+}$ ATPase on the basolateral membrane of the NPE, providing a likely mechanism for active transport of sodium out of the ciliary epithelium (see [32,40,59]). Extracellularly directed potassium and chloride channels as well as glutamate uptake and glutamine efflux transporters have also been identified on the basolateral membrane of the NPE (see [32,37,45,50]). While the identification of these ion transporters and channels advances the understanding of AH production, there are still many questions to be answered. Aside from the lack of consensus over a single conceptual model of ion transport across the ciliary epithelium, little is understood about the regional differences in ion transport over the entire span of the ciliary body. McLaughlin et al. propose that the anterior aspect of the ciliary epithelium is the primary site of aqueous humor secretion [23]. Other findings of the presence of the Na${}^{+}$/K${}^{+}$ ATPase on the basolateral membrane of the PE and Na${}^{+}$/K${}^{+}$/2Cl${}^{-}$ cotransporter on the basolateral membrane of the NPE suggest a potential reabsorptive role of the ciliary epithelium (see [41,53]). In addition, while aquaporin proteins have been identified as a means of facilitated diffusion of water across the NPE (see [33,49]), the effects of previously identified swelling activated K${}^{+}$ channels and osmosensors such as transient receptor potential vanilloid isoform 4 is unclear (see [46,51]). More recent findings of ENaC channels in the ciliary epithelial membrane and hemichannels, as well as inwardly rectifying K${}^{+}$ channels located on the basolateral membrane of the NPE (see [35,47,52]), demonstrate that there are more discoveries to be made to fully understand the interplay between ion transporters and channels in the ciliary epithelium.

### 1.4. Connecting AH Flow, Ocular Physiology, and Pathology: The Role of Mathematical Modeling

The flow of AH and its regulation play an important role in ocular physiology by contributing to the control of the IOP level [5,6]. Elevated IOP is the only approved treatable risk factor in glaucoma, an optic neuropathy characterized by a multifactorial etiology with a progressive degeneration of retinal ganglion cells that ultimately leads to irreversible vision loss [7,8]). Currently, glaucoma affects more than 60 million people worldwide and is estimated to reach almost 80 million by 2020 [60]. IOP can be lowered via hypotonizing eye drops and/or surgical treatment, and it can be shown that reducing IOP by 1mmHg has the effect of reducing the risk of glaucoma progression and subsequent vision loss by 10% [61]. As lowering AH production leads to lower IOP levels, the modulation of ionic secretion in the ciliary epithelium is an important pharmacological target for glaucoma therapies. In this perspective, it would be very useful to have a quantitative approach capable of relating changes in ionic secretion at the cell level with changes in AH flow and IOP at the eye level. Such an approach can be achieved by integrating experimental data with mathematical modeling of the fundamental mechanisms governing ocular physiology across length scales. Ultimately, this approach could serve as a virtual laboratory where drug responses could be tested in silico under various conditions, thereby aiding the design of more targeted and cost-effective experimental and clinical studies. A first step towards this ambitious goal is the model verification, which consists in addressing the question of whether the model succeeds in predicting known physiological conditions inside the CE cells once the configuration of ion exchangers on the CE membrane and the biochemical reactions inside the CE cells are known. As discussed in Section 1.3, however, the configuration of ion exchangers is not yet completely known, as a consensus is lacking across experimental studies. Thus, the first step of model verification yields a relevance on its own, as it can help interpret experimental findings by quantifying the implications that different choices for the configurations of ion exchangers would have on the CE physiology. Model verification is the goal of the present work, where, specifically, we utilize model simulations to:

**A1**  verify that the value of the predicted transepithelial potential difference is physiologically correct;
**A2**  verify that the values of the predicted intracellular ion concentrations are physiologically correct;
**A3**  verify that the value of the predicted NPE transmembrane potential difference is physiologically correct.

We notice that transepithelial potential difference and intracellular ion concentrations are characteristic of the cell level in the scheme of Figure 1 whereas the NPE transmembrane potential difference is characteristic of the membrane level in the scheme of Figure 1. In this sense, we see that our theoretical description of AH production is a genuinely multiscale/multiphysics formulation since it incorporates knowledge of very diverse physical processes occurring at very diverse length scales.

## 2. Results

**Figure 3:**
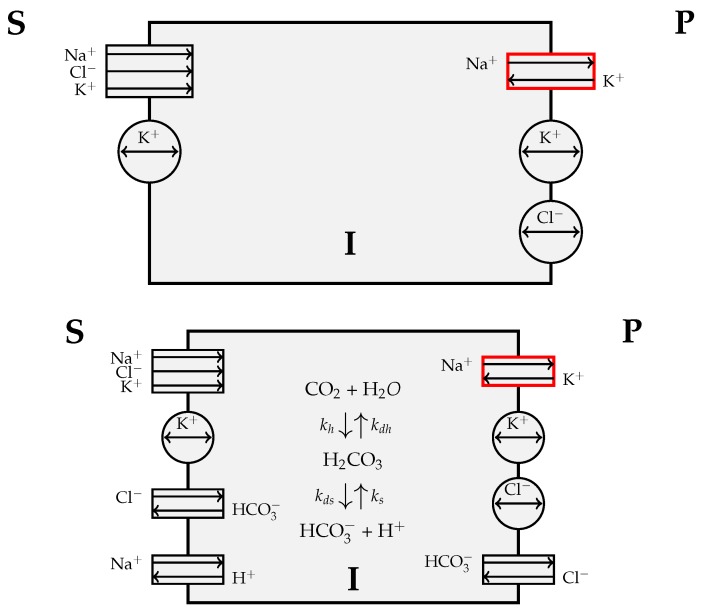
(**Top panel**) Schematic representation of a single ciliary epithelium (CE) cell and ion transporters in the case of Model 0. (**Bottom panel**) Schematic representation of a single CE cell and ion transporters in the case of Model 1.

In this section, we first illustrate the two configurations that have been used in the theoretical description of the PE/NPE cell couplet [17], and then, we show the principal results obtained by numerically solving the mathematical model described in Section 4. In both configurations, we consider the PE/NPE cell couplet as a single CE cell, which is constituted by the union of a PE cell and an NPE cell separated by gap junctions, as represented in the scheme of Figure 2. However, for the sake of simplifying the analysis of the problem, the presence of the gap junctions is not accounted in the model description of the cell which is thus regarded as a homogeneous compartment, endowed with two membranes. One membrane, located on the right side of the CE cell, separates the CE cell from the posterior chamber (denoted by **P**). The other membrane, located on the left side of the CE cell, separates the CE cell from the stroma (denoted by **S**). The intracellular region is denoted by **I**. Passive transporters are represented with circles, secondary active transporters with rectangles and primary active transporters with red border rectangles. The double head arrows in potassium and chloride uniporters indicate that the flow of ions depends only on the electrochemical gradient and not on active mechanisms. In all other transporters, the single arrows denote the selected direction of the flow of each species. In the center of the scheme of the CE cell used in Model 1 (bottom panel of Figure 3), the chemical reaction involving the CO${}_{2}$/HCO${}_{3}^{-}$ buffer is represented. The dissociation of CO${}_{2}$ into HCO${}_{3}^{-}$ is a two-step process that begins with the conversion of CO${}_{2}$ and water into carbonic acid (H${}_{2}$CO${}_{3}$). The interconversion of CO${}_{2}$ and water into H${}_{2}$CO${}_{3}$ is catalyzed by a family of enzymes known as carbonic anhydrases which greatly increases the reaction rate [62].

### 2.1. Model 0

The first configuration considered in this study henceforth referred to as Model 0, is schematically represented in the top panel of Figure 3. The following types of ion transporters are assumed to be present on the S/CE membrane:

1. Na${}^{+}$-K${}^{+}$-Cl${}^{-}$ symporter;
2. K${}^{+}$ uniporter.

The following types of ion transporters are assumed to be present on the CE/P membrane:

1. Na${}^{+}$-K${}^{+}$ pump;
2. K${}^{+}$ uniporter;
3. Cl${}^{-}$ uniporter.

### 2.2. Model 1

The second configuration considered in this study henceforth referred to as Model 1, is schematically represented in the bottom panel of Figure 3. The following types of ion transporters are assumed to be present on the S/CE membrane:

1. Na${}^{+}$-K${}^{+}$-Cl${}^{-}$ symporter;
2. K${}^{+}$ uniporter;
3. Cl${}^{-}$-HCO${}_{3}^{-}$ antiporter;
4. Na${}^{+}$-H${}^{+}$ antiporter.

The following types of ion transporters are assumed to be present on the CE/P membrane:

1. Na${}^{+}$-K${}^{+}$ pump;
2. K${}^{+}$ uniporter;
3. Cl${}^{-}$ uniporter;
4. Cl${}^{-}$-HCO${}_{3}^{-}$ antiporter.

We notice that, unlike Model 0, the configuration of Model 1 accounts for the intracellular interconversion between carbon dioxide and water into carbonic acid catalyzed by the carbonic anhydrase enzyme. The values of the bicarbonate concentrations in the stromal side of the CE and in the posterior chamber side of the CE are taken equal to 27 mM and 20 mM, respectively.

### 2.3. Verification of Model Predictions against Experimental Data

Let *X* denote any compartment in the schemes of Figure 3 (X=S: ciliary stroma, X=I: cell couplet intracellular region and X=P: posterior chamber). Let us also denote by $\varphi_{X}$ and $n_{s}^{X}$ the electric potential (expressed in mV) and ion concentration (expressed in mM) in the compartment *X*, respectively. The following benchmark quantities are used to compare model predictions with physiological available data:

- transepithelial potential difference $\varphi_{S}-\varphi_{P}$ (in mV);
- intracellular concentrations of Na${}^{+}$, K${}^{+}$, and Cl${}^{-}$ (in mM);
- NPE transmembrane potential difference $\varphi_{I}-\varphi_{P}$ (in mV).

#### 2.3.1. Results for Model 0

Table 3 contains the values of transepithelial potential difference $\varphi_{S}-\varphi_{P}$, intracellular concentrations of Na${}^{+}$, K${}^{+}$, Cl${}^{-}$, and NPE transmembrane potential difference $\varphi_{I}-\varphi_{P}$ predicted by numerically running Model 0.

#### 2.3.2. Results for Model 1

Table 4 contains the values of transepithelial potential difference $\varphi_{S}-\varphi_{P}$, intracellular concentrations of Na${}^{+}$, K${}^{+}$, Cl${}^{-}$, and NPE transmembrane potential difference $\varphi_{I}-\varphi_{P}$ predicted by numerically running Model 1.

## 3. Discussion

This section is divided into three parts. The first part contains a discussion of the predictions of Model 0. The second part contains a discussion of the predictions of Model 1. The third part contains a comparison between the predictions of the two models.

### 3.1. Discussion of the Predictions of Model 0

Model predictions listed in Table 3 show that only chloride concentration (10 mM) does not deviate too much from the range of measurements ([41,51] mM). Predicted potassium (55 mM) is much smaller than measured ([148,176] mM) whereas predicted sodium (75 mM) is much larger than measured ([12,18] mM). The predicted potential drop $\varphi_{S}-\varphi_{P}$ across the cell couplet is negative (−2 mV) instead of being positive as in measurements ([1.084,1.284] mV). However, the order of magnitude does not appreciably deviate from the measured range. The predicted transepithelial nonpigmented potential drop $\varphi_{I}-\varphi_{P}$ (−70 mV) agrees well with the lower bound for measured data ([−70,−50] mV).

Results show that all computed ion concentrations are positive but, in general, quite out of the measured range in the case of cations. This suggests that Model 0 does not provide a sufficiently accurate picture of the physiological conditions in the intracellular region of the CE cells, possibly because of the lack of chemical reactions mediated by the carbonic anhydrase enzyme. Simulated potential drops are reasonably accurate as far range as concerned, whereas the predicted sign of the potential drop matches the measured sign only in the case of the drop across the cell couplet (the cellular level). This suggests that Model 0 does not provide a sufficiently accurate picture of the NPE membrane, possibly because of the lack of secondary active transporters that control the exchange of anions associated with the intracellular interconversion between carbon dioxide and water into carbonic acid catalyzed by the carbonic anhydrase enzyme. These considerations are the basis for the development of Model 1 and the analysis of its predictions is conducted in the next section.

### 3.2. Discussion of the Predictions of Model 1

The computational complexity of Model 1 is significantly higher than that of Model 0, but this additional effort is fairly compensated by the outcome of simulations.

Model predictions listed in Table 4 show that predicted potassium (132.09 mM) is close to measured ([148,176] mM) and predicted sodium (15.59 mM) is very accurate since it lies at the center of the measured range ([12,18] mM). Predicted chloride concentration (almost 4 mM) is far smaller than the measured range ([41,51] mM). The predicted potential drop $\varphi_{S}-\varphi_{P}$ across the cell couplet is still negative (−1.6 mV) but closer to the measured range ([1.084,1.284] mV) than with Model 0. The predicted transepithelial nonpigmented potential drop $\varphi_{I}-\varphi_{P}$ (−93 mV) is instead more negative than measured data ([−70,−50] mV).

Results show that predicted intracellular cation concentrations are remarkably accurate whereas the accuracy of the predicted anion concentration is worse than in Model 0. This suggests that having included the chemical reactions mediated by the carbonic anhydrase enzyme has significantly increased the biophysical quality of the model but that, likely, a more careful characterization of the parameter values in the source term (15)–(18) has to be conducted. The simulated potential drop across the cell couplet is closer to the measured range than predicted by Model 0, but its sign is still opposite to what was measured. The simulated potential drop across the NPE membrane is more negative than predicted by Model 0, although it does not significantly deviate from measurements. The enhanced NPE depolarization predicted by Model 1 is likely due to anion accumulation inside the cell couplet associated with the increase of impermeant protein concentration required to restore electroneutrality. This suggests that other cation transporters may be needed in the theoretical description of the NPE membrane to improve the accuracy of the prediction of the electric variables. A possible extension could be to include the calcium-sodium and sodium-proton exchangers considered in the continuum-based model investigated in [18].

### 3.3. Comparison between Model 0 and Model 1

Summarizing the analysis of the previous two sections, we see that simulation results suggest that:

- the configuration of ion exchangers and the biochemical reactions mediated by the carbonic anhydrase enzyme strongly influence the prediction of intracellular ion concentrations and transmembrane potentials;
- Model 1 provides better estimates of intracellular concentrations than predicted by Model 0;
- the transmembrane potentials estimated by Models 0 and 1 are both within the same order of magnitude as those measured experimentally, but not exactly within the experimental range.

## 4. Materials and Methods

In this section, we provide a detailed description of various elements that allow us to construct the mathematical model for the simulation of the AH production in the CE.

### 4.1. Model Assumptions

In this section, we refer to the schemes illustrated in Figure 2 and Figure 3. Gap junctions link PE and NPE cells and provide low-resistance pathways interconnecting the intracellular fluids within and between the PE and NPE layers [21,22,65]. Experimental results also confirm that there are no significant variations in the electric potential across the gap junctions [29]. These observations lead one to introduce the following model assumptions:

- each pair of neighboring PE and NPE cells are considered as a single unit (referred to as the PE–NPE cell couplet) rather than two distinct compartments;
- ion transporters have uniform spatial distribution along the CE;
- the unit has a fixed volume;
- gap junctions within PE and NPE cells are neglected.

**Figure 4:**
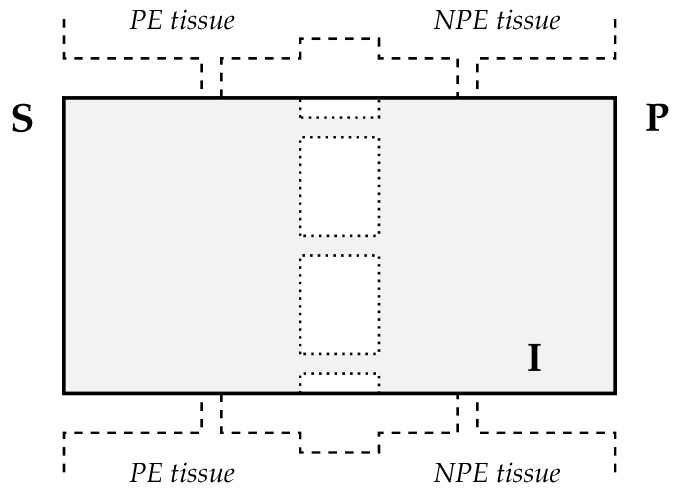
Schematic representation of PE–NPE cell couplet. The gap junctions are represented by the white rectangles with dashed border located in the center of the scheme.

A PE–NPE cell couplet is schematically represented in Figure 4 where we denote with **S**, **I**, and **P**, the stromal side, cytosol, and posterior chamber side, respectively.

### 4.2. Mathematical Model

The model presented in [66] is our starting point for deriving an ion secretion model for a cell formed by a PE–NPE couplet. The purpose is to find:

- electric potential at the **S** side and in the **I** region;
- ion concentrations in the **I** region,

starting from baseline experimental ion concentrations at sides **S** and **P**, and then compare the computed values with baseline experimental data. This comparison will be performed for Models 0 and 1, which differ by the configuration of ion exchangers and intracellular reactions. We emphasize that, while aiming at reproducing known physiological intracellular conditions, this study offers quantitative insights on the fundamental mechanisms that give rise to such conditions in the first place, thereby providing a valuable complement to experimental methods.

In both configurations of Model 0 and Model 1, we set $\varphi_{P}=0$ mV. This means that the **P** side of the cell couplet is electrically grounded. The unknowns of the problem in the case of Model 0 are:

- the electric potentials $\varphi_{S}$ and $\varphi_{I}$;
- the ion concentrations $n_{{\mathrm{Na}}^{+}}^{I}$, $n_{K^{+}}^{I}$, $n_{{\mathrm{Cl}}^{-}}^{I}$ and $n_{P^{-}}^{I}$.

The unknowns of the problem in the case of Model 1 are:

- the electric potentials $\varphi_{S}$ and $\varphi_{I}$;
- the ion concentrations $n_{{\mathrm{Na}}^{+}}^{I}$, $n_{K^{+}}^{I}$, $n_{{\mathrm{Cl}}^{-}}^{I}$, $n_{H^{+}}^{I}$, $n_{{\mathrm{HCO}}_{3}^{-}}^{I}$ and $n_{P^{-}}^{I}$;
- the concentrations $n_{{\mathrm{CO}}_{2}}^{I}$ and $n_{H_{2}{\mathrm{CO}}_{3}}^{I}$.

In both model configurations, $n_{P^{-}}^{I}$ is the intracellular concentration of an impermeant protein whose role is to enforce intracellular electroneutrality (see [66,67,68]).

Following [66], we write the balance equations for water volume and mass of each ionic species, electroneutrality within the **I** compartment and open-circuit conditions. We consider fluxes to be positive when entering the cell. Ion and neutral species are numbered as indicated in Table 5. The balance equations for Model 0 and Model 1 read:

(11) $$\begin{array}{ccc} \mathrm{Cell}\;\mathrm{volume}\;\mathrm{balance}: & \;S^{S}J_{V}^{S}+S^{P}J_{V}^{P}=0 & \end{array}$$

(12) $$\begin{array}{ccc} \mathrm{Solute}\;\mathrm{mass}\;\mathrm{balance}: & \;S^{S}J_{i}^{S}+S^{P}J_{i}^{P}+\Phi_{i}=0\;\;\forall i=1,\ldots ,N^{ion}\; & \end{array}$$

(13) $$\begin{array}{ccc} \mathrm{Electroneutrality}: & \;\sum_{i=1}^{N^{sp}}z_{i}n_{i}^{I}-n_{P^{-}}^{I}=0 & \end{array}$$

(14) $$\begin{array}{ccc} \mathrm{Open}\;\mathrm{circuit}\;\mathrm{conditions}: & \;\sum_{i=1}^{N^{sp}}z_{i}J_{i}^{S}=0 & \end{array}$$

where $N^{sp}$ denotes the number of chemical species considered in the model ($N^{sp}=3$ in the case of Model 0, $N^{sp}=7$ in the case of Model 0), $J_{V}^{S,P}$ (units: $\mu L\;\min^{-1}$) is the volumetric flow rate of water across the membrane at the **S** and **P** sides of the cell couplet, $J_{i}^{S,P}$ (units: $\mathrm{mM}\;m\;s^{-1}=\mathrm{mol}\;m^{-3}\;m\;s^{-1}=\mathrm{mol}\;m^{-2}\;s^{-1}$) is the mass flux density of the *i*–th ionic species, $\Phi_{i}$ (units: $\mathrm{mol}\;m^{-2}\;s^{-1}$) is equal to zero in the case of Model 0 whereas in the case of Model 1 it denotes the source term for the reacting solutes (H${}^{+}$, HCO${}_{3}^{-}$, CO${}_{2}$, H${}_{2}$CO${}_{3}$), $S^{S}$, $S^{P}$ are the membrane surface areas for unit of epithelium area, and $z_{i}$ is the valence of the *i*-th ion species (with $z_{i}=0$ in the case of neutral species). Extensive tests have shown that model predictions are very modestly influenced by the ratio $S^{S}/S^{P}$, so that in the remainder of the article we set $S^{S}=S^{P}=1$. In the case of Model 1, reaction terms are given by (see [66], Section 8.3.3):

(15) $$\begin{array}{cc} \; & \Phi_{{\mathrm{HCO}}_{3}^{-}}=-k_{s}n_{{\mathrm{HCO}}_{3}^{-}}^{I}n_{H^{+}}^{I}+k_{ds}n_{H_{2}{\mathrm{CO}}_{3}}^{I} \end{array}$$

(16) $$\begin{array}{cc} \; & \Phi_{{\mathrm{CO}}_{2}}=-k_{h}n_{{\mathrm{CO}}_{2}}^{I}+k_{dh}n_{H_{2}{\mathrm{CO}}_{3}}^{I} \end{array}$$

(17) $$\begin{array}{cc} \; & \Phi_{H^{+}}=\Phi_{{\mathrm{HCO}}_{3}^{-}} \end{array}$$

(18) $$\begin{array}{cc} \; & \Phi_{H_{2}{\mathrm{CO}}_{3}}=-\Phi_{{\mathrm{HCO}}_{3}^{-}}-\Phi_{{\mathrm{CO}}_{2}} \end{array}$$

where $k_{s}$, $k_{ds}$, $k_{h}$, and $k_{dh}$ are the association, dissociation, hydration, and dehydration constants, respectively, of the chemical reaction shown in the bottom panel of Figure 3. We notice that $k_{h}$ depends on the concentration of the carbonic anhydrase enzyme [62]. The values of the above constants are $k_{s}=10^{4}\;s^{-1}$, $k_{ds}=6.1\cdot 10^{4}\;s^{-1}$, $k_{h}=390\;s^{-1}$, and $k_{dh}=23\;s^{-1}$.

### 4.3. Numerical Solution and the Issue of Electroneutrality

To numerically solve the nonlinear system (11)–(14), we use the trust–region–reflective iterative algorithm implemented in MatLab through function lsqnonlin (see [69] and references cited therein), which is able to find the solution in a bounded region (in our case lower bounded since concentrations must be nonnegative). In the case of model 0, the initial guess of the solution algorithm is constituted by the values $n_{{\mathrm{Na}}^{+}}^{I,(0)}$, $n_{K^{+}}^{I,(0)}$ and $n_{{\mathrm{Cl}}^{-}}^{I,(0)}$ for the concentrations of sodium, potassium, and chloride in the intracellular region of the cell couplet, which have been set equal to 5 mM, 120 mM, and 25 mM, respectively. These data match experimental measurements (see [24]). However, the total (initial) intracellular ion concentration

(19) $$\begin{array}{ccc} \; & z_{{\mathrm{Na}}^{+}}n_{{\mathrm{Na}}^{+}}^{I,(0)}+z_{K^{+}}n_{K^{+}}^{I,(0)}+z_{{\mathrm{Cl}}^{-}}n_{{\mathrm{Cl}}^{-}}^{I,(0)}=5+120-25=100\;\mathrm{mM} & \end{array}$$

does not satisfy the constraint of electroneutrality inside the cell couplet cytosol

(20) $$\begin{array}{ccc} \; & \sum_{i}^{N_{ion}}z_{i}n_{i}^{I}=0. & \end{array}$$

Relation (20) must be satisfied by the ion concentrations predicted by the theoretical model because spatial variations of electric potential and ion concentrations are neglected in the electrochemical description of the system, so that Poisson’s equation, which mathematically expresses Gauss’ law at the differential level (see [25], Chapter 3), automatically degenerates into (20). Thus, comparing (19) with (20), we conclude that a large amount of negative ion concentration needs be introduced to counterbalance the excess of positive ion concentration inside the cell couplet. This negative ion concentration is provided by a large impermeant protein $P^{-}$ which plays the role of a “buffer” balancing any excess of positive ion concentration in the cell couplet (see [67]). A similar analysis can be conducted in the case of model 1. We refer to [68] for a thorough discussion of the issue of electroneutrality and its biophysical implications based on the asymptotic analysis of Poisson’s equation as a function of the scaled Debye length.

### 4.4. Models for Ion Fluxes

Ion flow across the cellular membrane depends on the type of ion transporter. In the following sections, we characterize the solute flux densities according to the classification of the various transporters illustrated in Section 2.1 and Section 2.2. Henceforth, $K_{B}=1.38\cdot 10^{-23}$ $m^{2}{\mathrm{Kgs}}^{-2}K^{-1}$, $N_{A}=6.022\cdot 10^{23}$ ${\mathrm{mol}}^{-1}$, $q=1.602\cdot 10^{-19}$ C, and T=310.15 K denote the Boltzmann constant, Avogadro constant, electron charge, and temperature, respectively, and $V_{th}=K_{B}T/q$ is the thermal voltage. The flux density of a solute *i* physically represents the amount of solute *i* (expressed in moles) that crosses a unit surface per unit time. A detailed characterization of all parameter values and related bibliographical sources can be found in [17].

#### 4.4.1. Uniporters

For uniporters, we use the Goldman–Hodgkin–Katz (GHK) model (see [25], Chapter 17). Upon introducing the dimensionless potential difference $\psi_{X}:=\left(\varphi_{X}-\varphi_{I}\right)/V_{th}$, X=S,P, the GHK expression of the uniporter flux density of solute *i* across the membrane separating the compartments **I** and **X** is

(21) $$\begin{array}{ccc} \; & J_{i}^{X}=-z_{i}h_{i}^{X}\psi_{X}\frac{n_{i}^{X}-n_{i}^{I}\;\exp\left(-z_{i}\psi_{X}\right)}{1-\exp(-z_{i}\psi_{X})}\; & \end{array}$$

where $h_{i}^{X}$ (units: ${\mathrm{ms}}^{-1}$) represents the permeability of the membrane between compartments **I** and **X** to the solute *i*. The values of the permeability for potassium and chloride are $h_{K^{+}}^{S}=h_{K^{+}}^{P}=39.14$ $\mathrm{cm}\;s^{-1}$ and $h_{{\mathrm{Cl}}^{-}}^{P}=40.6$ $\mathrm{cm}\;s^{-1}$.

#### 4.4.2. Antiporters and Symporters

To model antiporters and symporters, we use the non–equilibrium thermodynamic formalism (see [70] for an analogous description of renal tubular transport) and write the flux density of solute *i* in antiporters and symporters as

(22) $$\begin{array}{ccc} \; & J_{i}^{X}=\sum_{j=1}^{N^{transp}}L_{ij}RT\left(\ln\left(\frac{n_{j}^{X}}{n_{j}^{I}}\right)+z_{i}\psi^{X}\right) & , \end{array}$$

where $N^{transp}$ is the number of ionic species involved in the ion transporter and $L_{ij}$ (units: ${\mathrm{mol}}^{2}\;J^{-1}\;m^{-2}\;s^{-1}$) is a coefficient that couples the flux of solute *i* to the driving force exerted on species *j*.

#### 4.4.3. Pumps

Here we consider the sodium–potassium pump. The flux densities of sodium and potassium can be modeled as (see [66], Section 8.2.2):

(23) $$\begin{array}{cc} \; & {\left.J_{{\mathrm{Na}}^{+}}^{X}\right|}_{{\mathrm{Na}}^{+}K^{+}}=-J_{{\mathrm{Na}}^{+}}^{{\mathrm{Na}}^{+}K^{+},\;max}{\left(\frac{n_{{\mathrm{Na}}^{+}}^{I}}{n_{{\mathrm{Na}}^{+}}^{I}+Y_{{\mathrm{Na}}^{+}}^{I}}\right)}^{3}{\left(\frac{n_{K^{+}}^{X}}{n_{K^{+}}^{X}+Y_{K^{+}}^{X}}\right)}^{2} \end{array}$$

(24) $$\begin{array}{cc} \; & {\left.J_{K^{+}}^{X}\right|}_{{\mathrm{Na}}^{+}K^{+}}=-\frac{2}{3}{\left.J_{{\mathrm{Na}}^{+}}^{X}\right|}_{{\mathrm{Na}}^{+}K^{+}} \end{array}$$

where $J_{{\mathrm{Na}}^{+}}^{{\mathrm{Na}}^{+}K^{+},\;max}$ is the maximum efflux of sodium ions at steady-state (units: $\mathrm{mM}\;m\;s^{-1}$) whereas $Y_{{\mathrm{Na}}^{+}}^{I}$ and $Y_{K^{+}}^{X}$ are the apparent dissociation constants (units: mM).

### 4.5. Model for Water Flux

Water flux across membrane *X* is given by

(25) $$\begin{array}{ccc} \; & J_{V}^{X}=L_{w}^{X}\sigma_{s}^{X}\left(\pi_{s}^{I}-\pi_{s}^{X}\right)+L_{w}^{X}\sigma_{p}^{X}\left(\pi_{p}^{I}-\pi_{p}^{X}\right)+L_{w}^{X}\left(\Pi^{I}-\Pi^{X}\right), & \end{array}$$

where $L_{w}^{X}$ (units: $\mu L\;{\mathrm{mmHg}}^{-1}\;\min^{-1}$) is the permeability to water of the membrane at side *X*, X=S,P. Water permeability at the stromal and posterior chamber sides of the cell couplet is set equal to the same value of 0.3 $\mu L\;\min^{-1}\;\mathrm{mmHg}$. This data does not come from experiments performed at the membrane scale level, rather, it is taken from [9] where it represents the ciliary epithelium permeability, a parameter related to the eye scale (macroscopic scale) and not the cellular scale of the CE. Water flow is assumed to exit the cell through aquaporins, while the contributions through membrane and ion transporters are negligible. Thus, in our model, we set the reflection coefficients of the *X* membrane $\sigma_{s}^{X}=\sigma_{p}^{X}=1$. Relation (25) is formally analogous to the expression of fluxes used in [9,71], but there is a difference in meaning: despite the similar physical phenomena, our expressions are modeling the flux density at the cellular scale while in [71] the characterization was made for flow at systemic/eye level. This means that some of the coefficients, in principle, could have different values even though the equations where they have been used have a similar mathematical form.

## 5. Conclusions and Perspectives

In this article, we have proposed a theoretical approach to link biophysical phenomena occurring at different length scales in the ciliary epithelium, which ultimately contributes to the production of aqueous humor. The reason at the basis of this contribution, which to the best of our knowledge is unique in the literature, is that the CE is the primary site of aqueous humor (AH) production, which results from the combined action of ultrafiltration and ionic secretion. As the modulation of ionic secretion is a fundamental target for drug therapy in glaucoma, the principal pathology causing blindness worldwide, it is of utmost importance to identify the main factors contributing to it. As no consensus exists on which ion transporters may be considered as the relevant players in CE physiology, we propose in the present article a theoretical approach that can complement experimental methods in characterizing how and to what extent they contribute to the electrochemical conditions of CE. In particular, we compare two model configurations of the CE, which differ by *(i)* type of transporters included for ion exchange across the epithelial membrane, and *(ii)* presence of intracellular production of carbonic acid mediated by the carbonic anhydrase enzyme. Results suggest that:

- ion exchanger configuration and intracellular biochemical reactions strongly influence the model prediction of intracellular ion concentrations and transmembrane potentials;
- one of the two configurations predicts sodium and potassium intracellular concentrations and transmembrane potential much more accurately than the other;
- predicted transmembrane potentials are within the same order of magnitude as those measured experimentally, but not exactly within the experimental range.

These results show the capability of the proposed theoretical approach to provide qualitative and quantitative insights on the physiological electrochemical conditions of the ciliary epithelium. Similar investigations could be performed on more complex configurations of ion exchangers, as inspired by experimental studies. In addition, models could be extended by accounting for the fact that the activity of particular ion transporters, pumps, and channels present in the membrane of PE and NPE epithelial cells may be influenced by neurohumoral mechanisms such as P2Y receptor-dependent, cAMP, or calcium-dependent pathways. For example, the dependence on the level of second messenger stimulation (cAMP) of ionic pumping and convective washout of ions by the movement of aqueous fluid across the CE bilayer may be taken care of by adapting to the present formulation the phenomenological description of the basolateral ion concentration proposed in [19]. A second example of model extension may be to introduce in the solute mass balance Equation (12) an individual expression level for each considered ion transporter/exchanger located on the stromal and posterior chamber sides of the CE bilayer. A third example is the introduction of changes of intracellular Ca${}^{2+}$ as there is growing evidence that these changes may have a significant impact on the alteration of CE membrane transport processes (see [6] and references cited therein). A further possible extension of the models proposed in the present work is to include the role of aquaporins in the water flux balance Equation (11). Finally, future developments of the proposed approach include the study of CE physiology in health and disease, the estimate of changes in AH production induced by specific drugs and a systematic screening of experimental data on ionic transport mechanisms in AH secretion available for various animal species to find the animal species (if any) capable of “best approximating” the human species.

## Figures and Tables

**Table 1:** This table collects the data for the ciliary epithelium taken from [19] that are needed to evaluate the various fluxes in Equation (9).

Parameter	Value	Units
$L_{p}$	$2\cdot 10^{-5}$	$\mathrm{mL}\;\min^{-1}\;\mathrm{mmHg}$
$\Pi^{C}$	25	mmHg
$\Pi^{P}$	15	mmHg
$\sigma_{p}$	0.999	−
$\pi_{p}^{C}$	20	mmHg
$\pi_{p}^{P}$	0.11	mmHg
$L_{s}$	$1.53445\cdot 10^{-4}$	$\mathrm{mL}\;\min^{-1}\;\mathrm{mmHg}$
$n_{s}^{P}$	147	mM
$n_{s}^{S}$	145	mM

**Table 2:** This table collects experimental data on aqueous humor (AH) active secretion retrieved from the literature. The second column contains information on the species considered in the experiments. The third column contains the information on which ion exchangers are investigated whereas the fourth column contains the information on their location on the ciliary epithelium.

Study	Species	Transporter/Channel	Localization
[31]	Human	Gap junctions	Lateral surface of PE/NPE
			Apical junction of PE/NPE
[32]	Human	Na${}^{+}$/K${}^{+}$ ATPase	Basolateral membrane of NPE
		K${}^{+}$ channel	Basolateral membrane of NPE
[33]	Human	AQP1	NPE
[34]	Human	Na${}^{+}$/K${}^{+}$/2Cl${}^{-}$ cotransporter	Basolateral membrane of NPE
[35]	Human	ENaC	Unspecified
[36]	Bovine	Cl${}^{-}$/HCO${}_{3}^{-}$ exchanger	Basolateral membrane of PE
[37]	Bovine	Ca${}^{2+}$ dependent K${}^{+}$ channel	Basolateral membrane of NPE
		cAMP activated Cl${}^{-}$ channel	Basolateral membrane of NPE
[38]	Bovine	Cl${}^{-}$ dependent Na${}^{+}$/HCO${}_{3}^{-}$ cotransporter	Basolateral membrane of PE
[39]	Bovine	Na${}^{+}$/H+ exchanger	Basolateral membrane of PE
[40]	Bovine	Na${}^{+}$/K${}^{+}$ ATPase (alpha 1, 2, and 3 isoforms)	Basolateral membrane of NPE
[41]	Bovine	Na${}^{+}$/K${}^{+}$ ATPase (alpha 1 and beta 1 isoforms)	Basolateral membrane of PE
[42]	Bovine	Na${}^{+}$/K${}^{+}$/2Cl${}^{-}$ cotransporter	Basolateral surface of PE
[43]	Bovine	Na${}^{+}$/H+ exchanger	Basolateral membrane of PE
		Cl${}^{-}$/HCO${}_{3}^{-}$ exchanger	Basolateral membrane of PE
[44]	Porcine	Na${}^{+}$/K${}^{+}$ ATPase (alpha 1, 2, and 3 isoforms)	Basolateral membrane of NPE
		Tight junctions	Lateral membrane of NPE
[45]	Porcine	Na${}^{+}$/K${}^{+}$/2Cl cotransporter	Basolateral membrane of PE
		Cl${}^{-}$/HCO${}_{3}^{-}$ exchanger	Basolateral membrane of PE
		Cl${}^{-}$ channel	Basolateral membrane of NPE
[46]	Porcine	Swelling activated K${}^{+}$ channel	PE basolateral membrane
[47]	Porcine	Hemichannels	Basolateral membrane of NPE
[48]	Porcine	Na${}^{+}$/K${}^{+}$ exchanger	Basolateral membrane of NPE
[49]	Murine	APQ4	Basolateral membrane of NPE
[50]	Murine	Glutamate transporter	Basolateral membrane of NPE
		Glutamine transporter	Basolateral membrane of NPE
[51]	Murine	TRPV4 channel	NPE
[52]	Murine	Inwardly rectifying K${}^{+}$ channel	NPE
[53]	Leporine	Na${}^{+}$/K${}^{+}$/2Cl${}^{-}$ cotransporter	Basolateral membrane of NPE
[54]	Rabbit	Na${}^{+}$/K${}^{+}$ ATPase (alpha 2 and beta 3 isoforms)	Basolateral membrane of NPE
[55]	Porcine	Cx isoforms	Gap junctions between PE and NPE cells
[56]	Rabbits	Na${}^{+}$/K${}^{+}$ ATPase (alpha 2 isoform)	Basolateral membrane of NPE
[57]	Bovine	Na${}^{+}$/K${}^{+}$/2Cl cotransporter	Basolateral membrane of PE
		Cl${}^{-}$/HCO${}_{3}^{-}$ and Na${}^{+}$/H+ exchangers	Basolateral membrane of PE
		Cl${}^{-}$ Channel	Basolateral membrane of NPE
[58]	Rabbits	Na${}^{+}$/K${}^{+}$/2Cl${}^{-}$ cotransporter	Basolateral membrane of PE
		Cl${}^{-}$/HCO${}_{3}^{-}$ exchanger	Basolateral membrane of PE
		Cl${}^{-}$ channel	Basolateral membrane of NPE

**Table 3:** This table contains a summary of the predictions of Model 0 and of the corresponding physiological reference values.

Quantity	Model Prediction	Reference Range	Species	Bibliographical Source
$\varphi_{S}-\varphi_{P}$	−2 mV	[1.084,1.284] mV	Humans	[63]
$n_{{\mathrm{Na}}^{+}}^{I}$	75 mM	[12,18] mM	Rabbit	[64]
$n_{K^{+}}^{I}$	55 mM	[148,176] mM	Rabbit	[64]
$n_{{\mathrm{Cl}}^{-}}^{I}$	10 mM	[41,51] mM	Rabbit	[64]
$\varphi_{I}-\varphi_{P}$	−70 mV	[−70,−50] mV	Shark	[29]

**Table 4:** This table contains a summary of the predictions of Model 1 and of the corresponding physiological reference values.

Quantity	Model Prediction	Reference Range	Species	Bibliographical Source
$\varphi_{S}-\varphi_{P}$	−1.6 mV	[1.084,1.284] mV	Humans	[63]
$n_{{\mathrm{Na}}^{+}}^{I}$	15.59 mM	[12,18] mM	Rabbit	[64]
$n_{K^{+}}^{I}$	132.09 mM	[148,176] mM	Rabbit	[64]
$n_{{\mathrm{Cl}}^{-}}^{I}$	3.97 mM	[41,51] mM	Rabbit	[64]
$\varphi_{I}-\varphi_{P}$	−93 mV	[−70,−50] mV	Shark	[29]

**Table 5:** Numbering of ion and neutral species.

*i*	1	2	3	4	5	6	7
species	Na${}^{+}$	K${}^{+}$	Cl${}^{-}$	H${}^{+}$	HCO${}_{3}^{-}$	CO${}_{2}$	H${}_{2}$CO${}_{3}$

## Abbreviations

The following abbreviations are used in this manuscript:

CE	Ciliary Epithelium
AH	Aqueous Humor
IOP	Intraocular Pressure
PE	Pigmented Epithelial Cells
NPE	Nonpigmented Epithelial Cells
S	Stroma
P	Posterior Chamber
I	Intracellular region of cell couplet
cAMP	Cyclic adenosine monophosphate
